# Cross-scale genetic, molecular, and cellular triangulation prioritizes *ACSL5* as a T3-responsive candidate linking hypothyroidism liability to anemia

**DOI:** 10.3389/fendo.2026.1943954

**Published:** 2026-10-08

**Authors:** Hui Li, Tao Jiang, Huan Wang, Mei Wang, Sixi Xie

**Affiliations:** Department of Hematology, The People’s Hospital of Jianyang City, Jianyang, Sichuan, China

**Keywords:** ACSL5, anemia, causal inference, DNA methylation, erythroid differentiation, gene expression, hypothyroidism

## Abstract

**Purpose:**

Anemia is a recognized complication of hypothyroidism, but disease-level genetic studies have not identified a clear erythroid cell-intrinsic target. We integrated genetic validation, molecular-QTL prioritization, and replicated triiodothyronine (T3)-responsive erythroid experiments to identify candidates linking hypothyroidism liability to anemia.

**Materials and methods:**

Two-sample Mendelian randomization (MR) assessed genetically predicted hypothyroidism and anemia. Source metadata were reviewed for possible sample overlap, and two cross-biobank forward validations were performed. Strict reverse MR used the original OpenGWAS identifiers; because anemia provided no genome-wide-significant instruments, a *post hoc* analysis used *p* < 5 × 10^−6^. Non-major histocompatibility complex (MHC) methylation summary-based Mendelian randomization (SMR) and whole-blood expression SMR were integrated, followed by a public fine-mapping/colocalization lookup for two acyl-CoA synthetase long-chain family member 5 (*ACSL5*) cytosine-phosphate-guanine (CpG) sites and the *ACSL5* eQTL. *ACSL5* was examined in three independent hemin-induced K562, a human chronic myelogenous leukemia cell line experiments with or without T3; Triacsin C and oleic acid-bovine serum albumin (BSA) served as pathway-level perturbations.

**Results:**

Primary inverse-variance weighted (IVW) analysis associated hypothyroidism liability with higher anemia odds (OR = 1.0304; 95% CI, 1.0195–1.0414; *p* = 3.42 × 10^−8^). Both primary genome-wide association studies (GWASs) were UK Biobank-derived, and their exact participant overlap could not be determined from public summary-level information. A participant-independent FinnGen hypothyroidism-to-UK Biobank anemia validation reproduced the positive direction (28 instruments; IVW beta = 0.001448, *p* = 4.14 × 10^−4^); the complementary UK Biobank hypothyroidism-to-FinnGen anemia analysis was also positive. Strict original-ID reverse MR was not estimable; the exploratory eight-instrument analysis was null and heterogeneous. Six non-MHC CpGs passed Bonferroni correction, including two mapped to *ACSL5*, while whole-blood *ACSL5* expression showed an inverse SMR estimate. The public lookup returned no pairwise CpG-expression colocalization. In all three K562 experiments, hemin plus T3 increased *HBA1*, *HBA2*, *GYPA*, and *ACSL5* expression. Triacsin C suppressed erythroid-marker expression, whereas oleic acid-BSA produced a partial increase without exogenous T3.

**Conclusion:**

Cross-scale evidence prioritizes *ACSL5* as a T3-responsive candidate linking hypothyroidism liability to anemia and implicates long-chain fatty-acid handling as a research direction. However, negative CpG-expression colocalization and nongene-specific pharmacological interventions preclude a mechanistic conclusion. Direct *ACSL5* perturbation and primary erythroid-cell validation are required.

## Introduction

Anemia is common in hypothyroidism, but its causes are heterogeneous. Reduced erythropoietin stimulation, diminished marrow activity, iron or vitamin deficiency, and associated autoimmunity are well recognized (1, 2). Metabolic regulation within erythroid cells may add to these established systemic explanations.

Large cohorts tell a consistent story. In a pooled analysis of 16 cohorts with more than 42,000 participants, abnormal thyroid status was related to anemia; UK Biobank data likewise showed a higher anemia prevalence among participants with clinically diagnosed hypothyroidism (3, 4). Genetic analyses can extend those observational findings because they are less susceptible to many acquired clinical confounders.

Thyroid hormones act on erythropoiesis at several levels. They affect renal erythropoietin production, erythroid-progenitor proliferation and differentiation, oxygen demand, and nutrient handling (1, 2, 5). Mutations affecting thyroid hormone receptor alpha impair erythroid maturation (6), whereas low-nanomolar triiodothyronine (T3) enhances hemin-induced differentiation in K562 cells (7). These findings also suggest a contribution from thyroid-hormone-responsive transcription within erythroid cells.

Prior genetic studies are not directly interchangeable because thyroid exposures and hematologic outcomes differed. In the UK Biobank, clinical hypothyroidism showed an observational relationship with anemia, but genetically predicted thyrotropin and free thyroxine within the euthyroid range had no clear effect (4). A separate two-sample Mendelian randomization (MR) analysis linked autoimmune thyroid disease and overt hypothyroidism to pernicious anemia and selected erythropoietic traits (5). The present study focuses on genetic liability to hypothyroidism because it more closely matches the clinical question than small physiologic variation in circulating hormones.

Molecular QTL data add another layer. Summary-based Mendelian randomization (SMR) integrates genome-wide association study (GWAS) findings with methylation or expression QTL data and tests whether a molecular trait shares a genetic signal with the outcome (8). The heterogeneity in dependent instruments (HEIDI) test helps distinguish such patterns from associations driven by linked variants (8, 9). Here, methylation- and expression-based SMR were analyzed separately and combined only for candidate prioritization.

Acyl-CoA synthetase long-chain family member 5 (*ACSL5*) was selected for experimental follow-up without being designated the uniquely causal gene. It encodes an enzyme that converts long-chain fatty acids to acyl-CoA intermediates and can direct them toward beta-oxidation, phospholipid synthesis, triglyceride synthesis, and related routes (10, 11). These functions are relevant because erythroid maturation depends on membrane remodeling, heme and globin synthesis, mitochondrial adaptation, and redox control (12–14). Two *ACSL5*-associated CpG sites and the whole-blood expression result provided independent molecular signals, while *ZDHHC6* remained another multi-omics-prioritized candidate requiring future functional study.

Genome-wide methylation studies spanning thyroid disease and hypothyroidism provided the source literature for the predefined author-provided candidate list analyzed here (15–17). The source record sets contained 9,202 CpGs from Bisarro Dos Reis et al., 6,070 from Beltrami et al., and 6,991 from Cai and Hu (15–17). After collapsing repeated probe identifiers within and across the three sets, the panel contained 18,778 unique CpGs. We used “thyroid disease/hypothyroidism-related” as a source-based label for this predefined CpG panel; individual probes were not separately validated in hypothyroidism. A broader integrated genomic characterization of papillary thyroid carcinoma provides additional thyroid-disease context but was not used to define the candidate universe (18).

We used a cross-scale framework to connect disease-level hypothyroidism liability with an erythroid candidate suitable for experimental follow-up. The framework combined MR and cross-biobank validation, a predefined thyroid disease/hypothyroidism-related nonmajor histocompatibility complex (MHC) candidate CpG screen, expression SMR, a public colocalization check, and replicated K562 perturbation experiments. It was designed to prioritize a testable molecular hypothesis; establishing the complete T3-*ACSL5* mechanism requires direct follow-up.

## Materials and methods

### Study design

We organized the investigation in four stages. First, two-sample MR evaluated the relationship between genetic liability to hypothyroidism and anemia; source-provenance review, two cross-biobank forward validations, and original-ID reverse-direction analyses were then performed. Second, non-MHC methylation SMR and whole-blood expression SMR were compared to prioritize molecular candidates, followed by a *post hoc* public fine-mapping/colocalization lookup at *ACSL5*. Third, *ACSL5* was examined in three independent K562 experiments under T3 manipulation. Fourth, pathway-level perturbations tested whether altering long-chain fatty-acid activation or substrate supply changed erythroid-marker transcription. The MR component was reported in accordance with STROBE-MR recommendations (19).

### GWAS data and exposure-outcome MR analysis

GWAS exposure data were available for 473,703 European-ancestry participants (OpenGWAS identifier: ebi-a-GCST90029022). The anemia outcome (ukb-b-15790) included 463,010 European-ancestry participants and was defined by the secondary ICD-10 diagnosis code D64.9. Instruments met genome-wide significance (*p* < 5 × 10^−8^). Independence was enforced by clumping at a coefficient of determination (*R*^2^) < 0.001 within a 10,000-kb window. Harmonization retained 114 single-nucleotide polymorphisms (SNPs) for the primary analysis. Thirteen were palindromic, none were ambiguous, and every variant met the final keep criterion. Because the binary UK Biobank traits were analyzed using linear mixed models, the MR coefficients and derived odds ratios (ORs) describe GWAS-scale associations per unit of genetically predicted liability rather than individual-level risk ratios between diagnosed groups. OpenGWAS resources and harmonized summary-data workflows were accessed through MR-Base (20).

Instrument strength was calculated for each retained SNP as *F* = (β_exposure_/standard error (SE)_exposure_)^2^, with *F* > 10 considered evidence against weak-instrument bias. Variance explained was reconstructed separately for exposure and outcome as *R*^2^ = *F*/(*N* − 2 + *F*), and cumulative exposure *R*^2^ was approximated by summing the clumped per-SNP values. Directionality was then tested with Steiger analysis (21). **Supplementary Table S4** provides the full analysis record, including harmonized alleles, effects, standard errors, *p*-values, allele frequencies, sample sizes, *F* statistics, *R*^2^ values, Steiger results, and harmonization flags.

IVW was the primary estimator. MR-Egger regression, weighted median, simple mode, and weighted mode provided complementary estimates, all reported as ORs with 95% confidence intervals (CIs). Cochran’s *Q* assessed heterogeneity under IVW and MR-Egger, whereas the MR-Egger intercept tested directional pleiotropy (22). Mendelian Randomization Pleiotropy RESidual Sum and Outlier (MR-PRESSO) evaluated the global pleiotropy signal and searched for outlying instruments; where relevant, it also compared estimates before and after correction (23). Finally, leave-one-out analysis removed one SNP at a time to identify influential variants, following current sensitivity-analysis guidance (24).

### Assessment of sample overlap, independent replication, and reverse MR

We reviewed OpenGWAS metadata and the linked source publication for both primary GWAS identifiers. The exposure dataset was generated in the UK Biobank European-ancestry cohort using BOLT-LMM (25), and the outcome identifier is a UK Biobank D64.9 analysis. Participant identifiers, detailed sampling frames, and the actual participant intersection were unavailable; consequently, the extent of overlap could not be quantified from the public summary-level information. To assess whether the primary association persisted when exposure and outcome came from different biobanks, we performed two cross-biobank forward analyses. The main validation used FinnGen broad hypothyroidism (finn-b-HYPOTHYROIDISM; 26,342 cases and 59,827 controls) as exposure and the original UK Biobank anemia outcome as the outcome (26). Genome-wide-significant instruments were clumped at *R*^2^ < 0.001 within 10,000 kb; 33 instruments were extracted, and 28 were harmonized. A complementary analysis queried the 114 original UK Biobank hypothyroidism instruments against FinnGen R12 D3_ANAEMIA (37,924 cases and 111,976 controls); 113 were harmonized. Multiplicative random-effects IVW was primary, with MR-Egger and weighted median as complementary estimators. Coefficient magnitude was not compared across differently scaled binary-trait GWASs.

For reverse MR, we swapped the original identifiers: ukb-b-15790 was the exposure, and ebi-a-GCST90029022 was the outcome. The prespecified genome-wide significance threshold (*p* < 5 × 10^−8^), with clumping at *R*^2^ < 0.001 within 10,000 kb, returned no eligible anemia instruments, so strict reverse MR could not be estimated. To explore directionality only, we conducted a *post hoc* analysis at *p* < 5 × 10^−6^ using the same clumping parameters. Nine anemia instruments were extracted, eight were harmonized, and *F* statistics ranged from 21.28 to 29.49. IVW, MR-Egger, and weighted median were compared. The analysis was exploratory and not confirmatory because it used subgenome-wide-significant instruments, involved the same overlapping UK Biobank source studies, and was not prespecified.

### Methylation-outcome SMR analysis

The multiple-testing set was assembled from three published genome-wide methylation studies of thyroid disease or hypothyroidism (15–17). The source record sets contained 9,202, 6,070, and 6,991 CpGs from Bisarro Dos Reis et al., Beltrami et al., and Cai and Hu, respectively. Records were merged, and repeated entries were collapsed only by probe identifier, yielding 18,778 unique CpGs. Before calculating the denominator, we did not filter by source-study *p*-value, effect size, direction, genomic location, tissue, or statistical significance. Of these 18,778 CpG sites, 855 appeared in the methylation quantitative trait locus (mQTL)-SMR output. The MHC was excluded *a priori*. Removing probes within the hg19/GRCh37 interval chr6:28,477,797–33,448,354 eliminated 802 CpG sites and left 53 non-MHC sites. Significance required *p*_SMR_ < 2.663 × 10^−6^ (0.05/18,778). *p*_HEIDI_ > 0.01 was treated as compatible with a shared genetic effect under the prespecified rule; missing HEIDI results remained unclassified. The blood mQTL resource was derived from replicated QTL in the Brisbane Systems Genetics Study and Lothian Birth Cohorts (27).

### Gene-expression-outcome SMR analysis

Three genes were taken forward from the non-MHC methylation results: *ACSL5*, *ZDHHC6*, and *DPYSL4*. Targeted whole-blood expression quantitative trait locus (eQTL)-SMR analyses reported transcript-outcome ORs, 95% CIs, and *p*_SMR_ values. The HEIDI threshold remained *p*_HEIDI_ > 0.01. Results were interpreted as independent expression-outcome signals without inferring that the associated CpGs regulate gene expression. The eQTL data came from the Genotype-Tissue Expression Project (GTEx) (28).

### *Post hoc* ACSL5 CpG-expression colocalization lookup

We queried the public Human Genotype-Phenotype Map (GPMap) for the *ACSL5* locus on GRCh38 (29). The lookup included whole-blood GoDMC cis-methQTL signals for cg02402091 and cg18733757 and the GTEx v10 whole-blood *ACSL5* eQTL within the same European LD block. We examined the precomputed fine-mapping colocalization-group assignments and searched all pairwise records without imposing a minimum PP.H4 threshold. A returned pair was considered evidence of two extracted signals sharing a fine-mapped causal variant. When no pair was returned, we recorded an absence of positive colocalization support in this resource without inferring independence between the signals.

### Cell culture and experimental treatments

Cell identity was confirmed by STR profiling, and PCR-based mycoplasma testing was negative before experimentation. Cells were seeded at 2 × 10^5^ cells/mL in six-well plates containing phenol-red-free RPMI-1640 and 10% charcoal-stripped fetal bovine serum, thereby limiting background thyroid-hormone activity. Triacsin C was added at 1 μmol/L in DMSO. Oleic acid-BSA was supplied at 100 μmol/L oleate equivalent (oleate:BSA, 5:1). Hemin (20 μmol/L for 48 h) initiated erythroid-marker induction; the supplemented condition also contained 1 nmol/L T3. Five conditions were studied in each independent experiment: vehicle alone; hemin plus T3; hemin plus vehicle (no exogenous T3); hemin plus T3 and Triacsin C; and hemin plus vehicle and oleic acid-BSA. Triacsin C inhibits several long-chain acyl-CoA synthetase isoforms (30), whereas oleic acid-BSA altered substrate availability. At 1 μmol/L, Triacsin C was used as an exploratory pathway-level intervention. Biochemical target engagement and *ACSL5*-specific inhibition were not assessed because no dose-response or ACSL activity assay was performed. There were three biological experiments. Each contained three technical quantitative polymerase chain reaction (qPCR) wells per condition.

### RNA extraction, reverse transcription, and real-time quantitative PCR

Total RNA was extracted with TRIzol reagent (1596-026, Invitrogen (Life Technologies Corporation), Carlsbad, CA, USA). After lysis, chloroform was added, and the samples were centrifuged to separate the aqueous phase. RNA was then precipitated with an equal volume of isopropanol, washed sequentially with 75% and absolute ethanol, air-dried, and dissolved in 20 μL diethyl pyrocarbonate-treated water. Samples were stored at − 80 °C until reverse transcription.

Reverse transcription used a 25-μL reaction. It contained 12 μL RNA-primer mix, 5 μL 5× RT reaction buffer, 1 μL 25 mmol/L dNTPs, 1 μL RNase inhibitor (25 U/μL), 1 μL M-MLV reverse transcriptase (200 U/μL), 1 μL oligo(dT)18, and 4 μL nuclease-free water. Incubation proceeded at 42 °C for 60 min and 70 °C for 5 min. The laboratory record listed Fermentas K1622 as the reverse-transcription kit.

qPCR used SYBR Green chemistry (K0223, Thermo Fisher Scientific Baltics UAB, Vilnius, Lithuania) on an ABI 7300 Real-Time PCR System. Each 25-μL reaction contained 10 μL SYBR Green mix, 1 μL of each primer, 11 μL water, and 2 μL cDNA. Cycling began with 95 °C for 10 min, followed by 40 cycles at 95 °C for 15 s and 55 °C for 45 s. The dissociation program was 95 °C for 15 s, 60 °C for 1 min, 95 °C for 15 s, and 60 °C for 15 s. Data were processed in ABI Prism 7300 SDS Software. Glyceraldehyde-3-phosphate dehydrogenase (*GAPDH*) was the sole reference gene and was stable across the five conditions: cycle threshold (Ct) values ranged from 19.19 to 19.76 across 45 technical wells. qPCR methods were reported according to Minimum Information for Publication of Quantitative Real-Time PCR Experiments (MIQE) recommendations (31). **Table 1** lists the primer sequences and expected amplicon lengths.

**Table 1:** Primer sequences used for real-time quantitative PCR.

Gene	Reference sequence	Forward primer (5′-3′)	Reverse primer (5′-3′)	Amplicon
*HBA1*	NM_000558.5	GCCGACAAGACCAACGTCAAG	AGGTCGAAGTGCGGGAAGTAG	131 bp
*HBA2*	NM_000517.6	ACTTCCCGCACTTCGACCTGAG	TCACCAGCAGGCAGTGGCTTAG	198 bp
*GYPA*	NM_001308187.2	GGCAATGCACACTTCAACTTC	TCCAATAACACCAGCCATCAC	202 bp
*ACSL5*	NM_001387037.1	AAACCAAACCAGCCCTAC	GCAAAGATGCCGACAAAC	125 bp
*GAPDH*	NM_001256799.3	AATCCCATCACCATCTTC	AGGCTGTTGTCATACTTC	218 bp

The reference sequences, forward and reverse primer sequences, and expected amplicon lengths for *HBA1*, *HBA2*, *GYPA*, *ACSL5*, and *GAPDH* are listed.

### qPCR data processing and statistical analysis

*GAPDH* was the reference gene. For each technical well, ΔCt = Ct_target_ − Ct*_GAPDH_*, and normalized expression was reported as 2^-^ΔCt (32). Technical wells were averaged within each biological experiment; they were not analyzed as independent observations. Group values are the mean ± standard deviation (SD) of the three biological-replicate means. Overall condition effects were tested separately for each gene by one-way repeated-measures analysis of variance on mean ΔCt, with experiment as the matched factor and Greenhouse–Geisser correction. Four paired contrasts were prespecified. Two-sided paired *t*-tests compared hemin plus T3 with vehicle, hemin plus vehicle with hemin plus T3, hemin plus T3 plus Triacsin C with hemin plus T3, and oleic acid-BSA with hemin plus vehicle. Holm adjustment was used to control for multiple comparisons within each gene. Fold changes were calculated within each experiment from ratios of mean normalized expression and then summarized as mean ± SD. With only three biological replicates, *p*-values were interpreted alongside effect sizes and consistency across experiments. Holm-adjusted *p* < 0.05 defined statistical significance for the prespecified contrasts.

## Results

### Association of hypothyroidism liability with anemia risk

After harmonization, 114 SNPs entered the primary MR analysis. Instrument strength was adequate throughout: F statistics ranged from 28.84 to 777.18, and the summed observed-scale per-SNP *R*^2^ was 0.0187 (1.87%). Thirteen variants were palindromic, although none was ambiguous. The IVW estimate indicated higher anemia odds with greater genetic liability to hypothyroidism (OR = 1.0304; 95% CI, 1.0195–1.0414; *p* = 3.42 × 10^−8^). Weighted median (OR = 1.0247; 95% CI, 1.0077–1.0420; *p* = 0.00427), MR-Egger (OR = 1.0285; 95% CI, 1.0051–1.0524; *p* = 0.0182), and weighted mode (OR = 1.0300; 95% CI, 1.0078–1.0526; *p* = 0.00888) gave similar point estimates. The simple-mode result crossed the null (OR = 1.0311; 95% CI, 0.9971–1.0662; *p* = 0.0759). **Table 2** provides all method-specific estimates; **Figure 1A** displays the SNP-level associations and fitted lines.

**Table 2:** Mendelian randomization estimates for hypothyroidism and anemia.

Method	SNPs	OR	95% CI	*P*-value	Interpretation
Inverse-variance weighted	114	1.0304	1.0195–1.0414	3.42 × 10^−8^	Primary estimate
Weighted median	114	1.0247	1.0077–1.0420	0.00427	Directionally consistent
MR-Egger	114	1.0285	1.0051–1.0524	0.0182	Directionally consistent
Simple mode	114	1.0311	0.9971–1.0662	0.0759	Concordant; imprecise
Weighted mode	114	1.0300	1.0078–1.0526	0.00888	Directionally consistent

The primary and complementary MR estimates, including the number of SNPs, odds ratio, 95% confidence interval, *p*-value, and interpretation for each method, are summarized.

**Figure 1:**
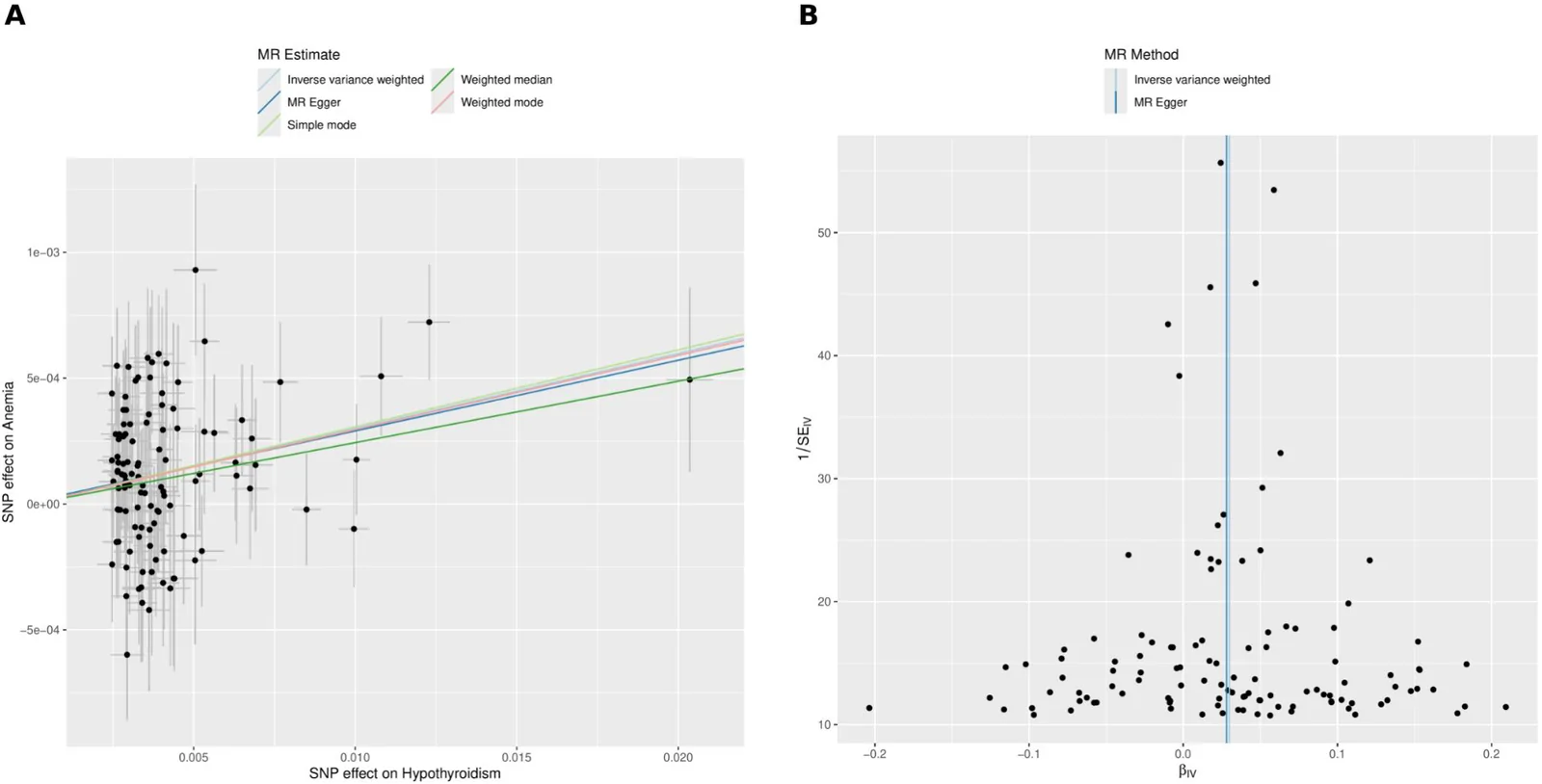
Scatter and funnel plots for the Mendelian randomization analysis of genetically predicted hypothyroidism and anemia. **(A)** Scatter plot showing SNP-specific associations with hypothyroidism on the *x*-axis and anemia on the *y*-axis. The fitted lines represent causal estimates obtained using inverse-variance weighted (IVW), MR-Egger, simple mode, weighted median, and weighted mode methods. **(B)** Funnel plot of SNP-specific causal estimates against their precision (1/SE), with vertical reference lines corresponding to the IVW and MR-Egger estimates.

Neither the IVW nor MR-Egger Q statistic indicated marked heterogeneity in the primary analysis (IVW: *Q* = 128.461, *df* = 113, *p* = 0.1518; MR-Egger: *Q* = 128.426, *df* = 112, *p* = 0.1374). Directional pleiotropy was not evident from the MR-Egger intercept (9.31 × 10^−6^; *p* = 0.8613). MR-PRESSO identified no outlier SNP, and sequential SNP removal left the IVW estimate largely unchanged. For every instrument, exposure-side *R*^2^ exceeded outcome-side *R*^2^; Steiger *p*-values ranged from 1.71 × 10^−77^ to 0.0208. The Steiger results supported the prespecified direction; reverse MR was retained as the formal directionality analysis. **Figure 1B** shows the funnel plot. Complete harmonized data are in **Supplementary Table S4**, with SNP-specific forest and leave-one-out analyses in **Supplementary Figure S1**.

### Sample-overlap assessment, cross-biobank validation, and reverse MR

Source verification confirmed that ebi-a-GCST90029022 and ukb-b-15790 were both UK Biobank-derived (33). Public summary-level information did not include the participant-level identifiers needed to calculate the exact intersection, so no numerical overlap estimate is reported. In the main cross-biobank validation, 28 FinnGen broad hypothyroidism instruments were harmonized with the original UK Biobank anemia outcome. The positive direction was reproduced by random-effects IVW (beta = 0.001448; SE = 0.000410; *p* = 4.14 × 10^−4^) and weighted median (beta = 0.001492; SE = 0.000558; *p* = 0.00755); MR-Egger was positive but less precise (beta = 0.002035; SE = 0.001104; *p* = 0.0765). Heterogeneity was not evident (IVW *Q* = 33.046; *df* = 27; *p* = 0.1956), and the MR-Egger intercept did not indicate directional pleiotropy (*p* = 0.5708). In the complementary UK Biobank hypothyroidism-to-FinnGen anemia analysis, 113 instruments were harmonized and all three estimators were positive: IVW beta = 3.838 (SE = 0.337; P = 5.58 × 10^−30^), MR-Egger beta = 4.331 (SE = 0.704; *p* = 1.24 × 10^−8^), and weighted-median beta = 3.772 (SE = 0.471; *p* = 1.14 × 10^−15^). Heterogeneity was present, but the MR-Egger intercept was not significant (*p* = 0.4262). Coefficient magnitudes are not directly comparable across different binary-trait GWAS scales; the relevant finding is replication of the positive direction when exposure and outcome came from different biobanks (**Supplementary Table S5**).

At *p* < 5 × 10^−8^, ukb-b-15790 supplied no eligible instruments, so strict original-ID reverse MR could not be estimated. In the *post hoc p* < 5 × 10^−6^ analysis, eight of nine extracted instruments were harmonized. Estimates were null across random-effects IVW (beta = 0.00497; SE = 0.22349; *p* = 0.982), MR-Egger (beta = − 0.17049; SE = 1.67177; *p* = 0.922), and weighted median (beta = − 0.04580; SE = 0.19239; *p* = 0.812). IVW heterogeneity was present (*Q* = 16.198; *df* = 7; *p* = 0.0234), whereas the MR-Egger intercept was not significant (*p* = 0.919). These exploratory results provide no evidence that genetic liability to anemia increases hypothyroidism risk. Given that no genome-wide-significant instruments were available and the relaxed analysis used overlapping source studies, reverse causation cannot be definitively excluded (**Supplementary Table S6**).

### Non-MHC methylation SMR signals

Among the 18,778 unique CpGs in the three-study thyroid disease/hypothyroidism-related panel, 855 appeared in the mQTL-SMR output. Excluding the prespecified MHC interval removed 802 probes and left 53 non-MHC CpGs (**Supplementary Table S1**). Six met the revised Bonferroni threshold of 2.663 × 10^−6^. Two were annotated to *ACSL5*; the remaining four comprised one *ZDHHC6*, one *JAKMIP3*, and two *DPYSL4* signals.

Both *ACSL5* probes were inverse. The estimates were cg18733757 (OR = 0.9985; 95% CI, 0.9979–0.9991; *p*_SMR_ = 1.38 × 10^−6^; *p*_HEIDI_ = 0.137) and cg02402091 (OR = 0.9983; 95% CI, 0.9976–0.9990; *p*_SMR_ = 1.54 × 10^−6^; *p*_HEIDI_ = 0.0118), and each had *p*_HEIDI_ > 0.01. *ZDHHC6* showed the same direction. Its probe cg15952994 also met the HEIDI criterion (OR = 0.9981; 95% CI, 0.9973–0.9988; *p*_SMR_ = 1.18 × 10^−6^; *p*_HEIDI_ = 0.529). HEIDI results were unavailable for *JAKMIP3* and *DPYSL4*, so those loci were treated as secondary, unclassified signals. **Figure 2** and **Table 3** display the full set of six Bonferroni-significant non-MHC CpGs.

**Figure 2:**
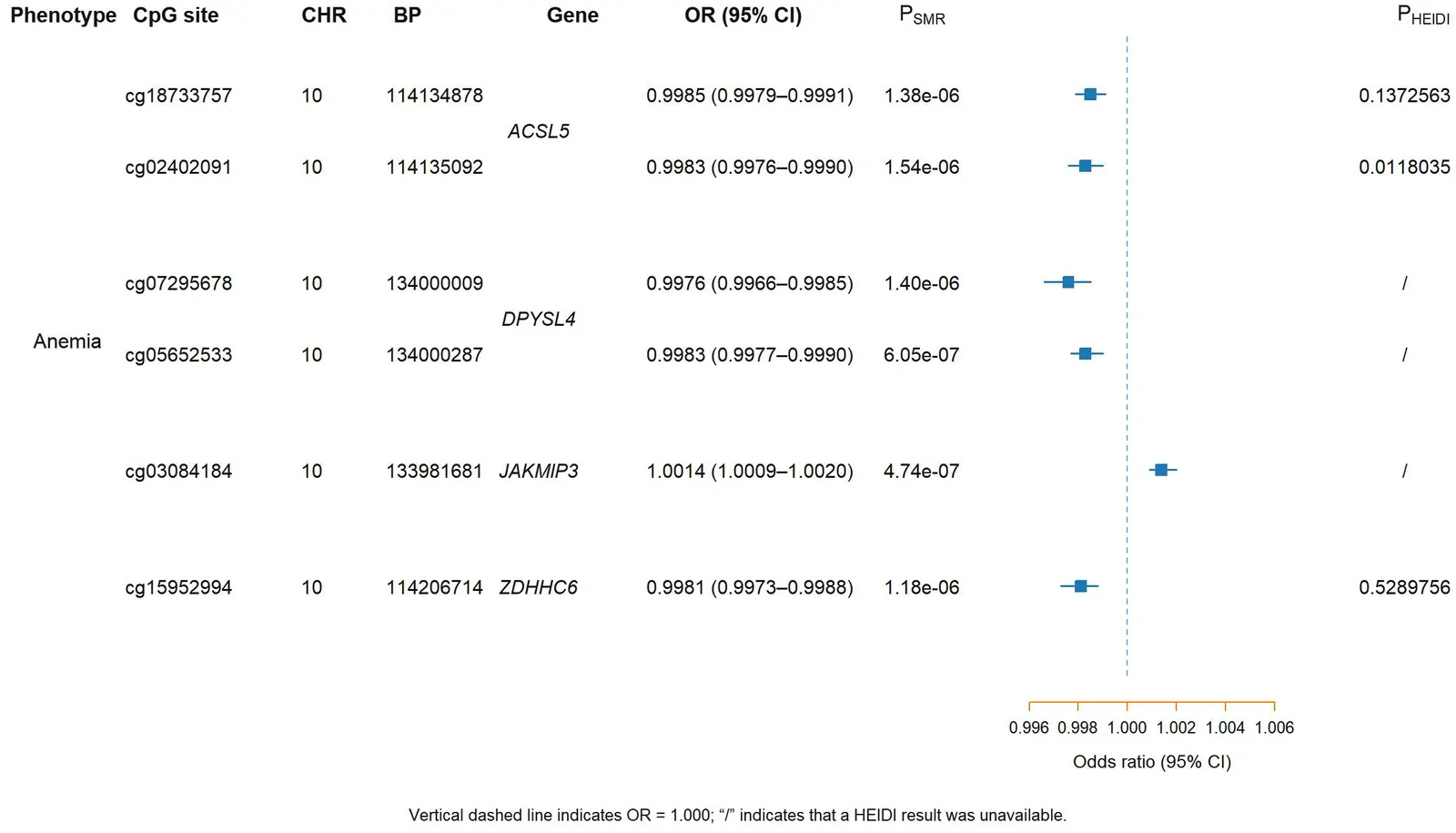
Forest plot of Bonferroni-significant non-MHC CpG sites in the methylation-outcome SMR analysis. Squares indicate odds ratios (ORs), horizontal lines indicate 95% confidence intervals (CIs), and the vertical reference line denotes OR = 1. The table columns report the CpG site, chromosome (CHR), base-pair position (BP), annotated gene, *p*_SMR_, and *p*_HEIDI_. The slash symbol indicates that a HEIDI result was unavailable.

**Table 3:** Bonferroni-significant non-MHC methylation SMR signals.

CpG	Gene	OR	95% CI	*p*_SMR_	*p*_HEIDI_
cg15952994	*ZDHHC6*	0.9981	0.9973–0.9988	1.18 × 10^−6^	0.529
cg18733757	*ACSL5*	0.9985	0.9979–0.9991	1.38 × 10^−6^	0.137
cg02402091	*ACSL5*	0.9983	0.9976–0.9990	1.54 × 10^−6^	0.0118
cg03084184	*JAKMIP3*	1.0014	1.0009–1.0020	4.74 × 10^−7^	Not available
cg07295678	*DPYSL4*	0.9976	0.9966–0.9985	1.40 × 10^−6^	Not available
cg05652533	*DPYSL4*	0.9983	0.9977–0.9990	6.05 × 10^−7^	Not available

The six non-MHC CpG sites passing the revised Bonferroni threshold (*p*_SMR_ < 2.663 × 10^−6^) are presented, with the annotated gene, odds ratio, 95% confidence interval, *p*_SMR_, *p*_HEIDI_, and HEIDI result. *p*_HEIDI_ > 0.01 indicates compatibility with a shared genetic effect under the prespecified threshold. Formal colocalization represents a complementary next-stage analysis.

*NA*, a HEIDI result was unavailable.

### Expression SMR findings for ACSL5

Whole-blood *ACSL5* expression yielded an inverse SMR estimate for anemia (OR = 0.9970; 95% CI, 0.9957–0.9983; *p*_SMR_ = 4.67 × 10^−6^; *p*_HEIDI_ = 0.0257). The two *ACSL5* methylation estimates and the expression estimate were all inverse with respect to anemia, but they remained independent associations and did not establish a methylation-to-expression pathway. *ZDHHC6* also showed an inverse expression-level estimate (OR = 0.9914; 95% CI, 0.9872–0.9956; *p*_SMR_ = 6.36 × 10^−5^; *p*_HEIDI_ = 0.0924). *DPYSL4* expression corresponded to higher anemia odds (OR = 1.0018; 95% CI, 1.0010–1.0026; *p*_SMR_ = 1.01 × 10^−5^); without a HEIDI result, it remained unclassified (**Supplementary Table S2**). Transcript-level results are shown in **Figure 3** and **Table 4**.

**Figure 3:**
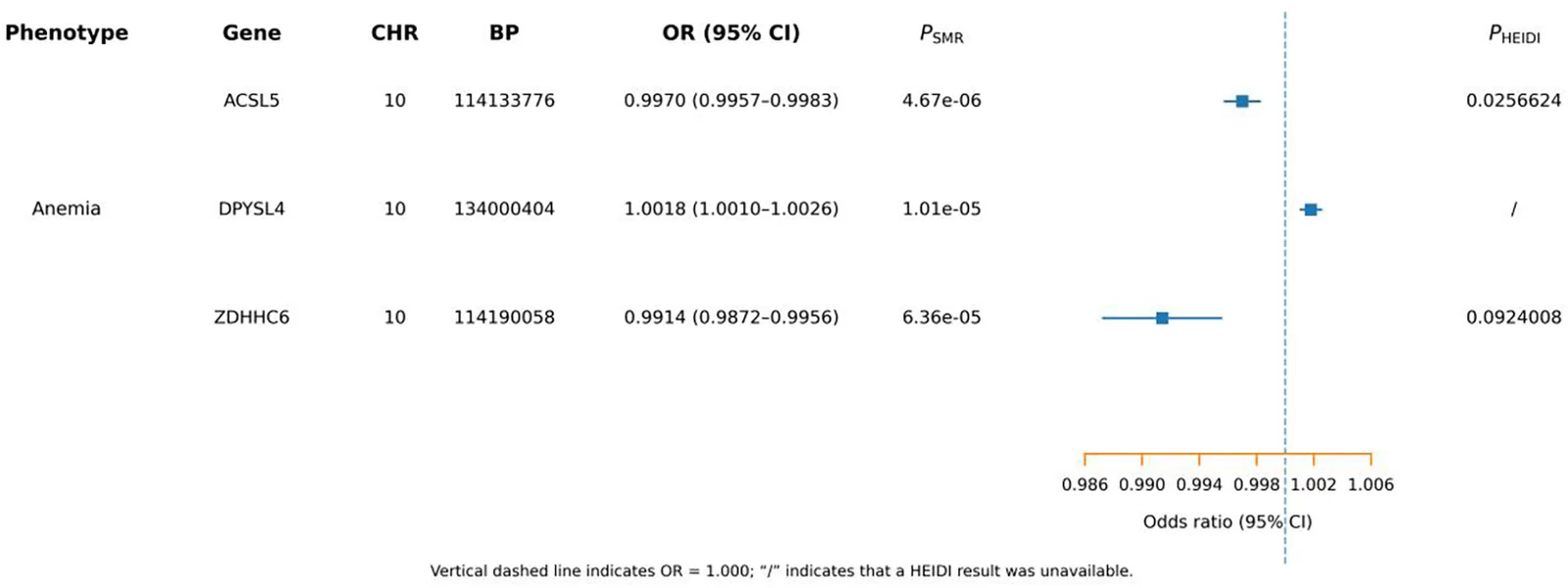
Forest plot of whole-blood gene-expression SMR results for anemia. Squares indicate odds ratios (ORs), horizontal lines indicate 95% confidence intervals (CIs), and the vertical reference line denotes OR = 1. The table columns report the phenotype, gene, chromosome (CHR), base-pair position (BP), *p*_SMR_, and *p*_HEIDI_. The slash symbol indicates that a HEIDI result was unavailable.

**Table 4:** Whole-blood gene-expression SMR results.

Gene	OR	95% CI	*p*_SMR_	*p*_HEIDI_
*ACSL5*	0.9970	0.9957–0.9983	4.67 × 10^−6^	0.0257
*ZDHHC6*	0.9914	0.9872–0.9956	6.36 × 10^−5^	0.0924
*DPYSL4*	1.0018	1.0010–1.0026	1.01 × 10^−5^	Not available

The targeted eQTL-SMR estimates for *ACSL5*, *ZDHHC6*, and *DPYSL4* are reported together with the HEIDI results. *p*_HEIDI_ > 0.01 indicates compatibility with a shared genetic effect under the prespecified threshold. Formal colocalization represents a complementary next-stage analysis.

*NA*, a HEIDI result was unavailable.

### *Post hoc* ACSL5 CpG-expression colocalization lookup

In GPMap, cg02402091 and cg18733757 were assigned to distinct whole-blood methQTL colocalization groups (groups 9284 and 9320, respectively), both represented by rs2419621. The GTEx v10 whole-blood *ACSL5* eQTL was assigned to group 9317, represented by rs1926565. No pairwise colocalization record connected either CpG extraction site to the *ACSL5* eQTL, even when all returned PP.H4 values were searched without applying a threshold. The public lookup provided no positive support for a shared fine-mapped causal signal. Methylation and expression SMR therefore remain separate evidence layers, with no demonstrated methylation-to-expression pathway (**Supplementary Table S7**).

### T3-responsive erythroid expression in K562 cells

Amplification was reproducible. All three experiments generated sigmoidal curves for hemoglobin subunit alpha 1 (*HBA1*), hemoglobin subunit alpha 2 (*HBA2*), glycophorin A (*GYPA*), *ACSL5*, and *GAPDH*, while *GAPDH* Ct values across the 45 technical wells remained within 19.19–19.76. Overall condition effects were detected for *HBA1* (Greenhouse–Geisser-adjusted *p* = 0.00273), *HBA2* (*p* = 0.000214), *GYPA* (*p* = 0.000728), and *ACSL5* (*p* = 0.000369). **Table 5** and **Figures 4A–D** summarize the normalized data; raw Ct values and normalized expression are provided in **Supplementary Table S3**.

**Table 5:** Normalized qPCR expression across three independent biological experiments.

Condition	*HBA1*	*HBA2*	*GYPA*	*ACSL5*
K562 + vehicle	0.1513 ± 0.0808	0.0538 ± 0.0141	0.1108 ± 0.0255	0.2326 ± 0.0796
K562 + hemin + T3	0.4207 ± 0.2214	0.1449 ± 0.0375	0.2740 ± 0.0648	0.4967 ± 0.1985
K562 + hemin + vehicle	0.2376 ± 0.1280	0.0787 ± 0.0193	0.1655 ± 0.0376	0.2683 ± 0.1197
K562 + hemin + T3 + Triacsin C	0.2038 ± 0.1156	0.0707 ± 0.0207	0.1513 ± 0.0382	0.4599 ± 0.1920
K562 + hemin + vehicle + oleic acid-BSA	0.3110 ± 0.1497	0.1067 ± 0.0335	0.2186 ± 0.0513	0.2857 ± 0.1308

**Figure 4:**
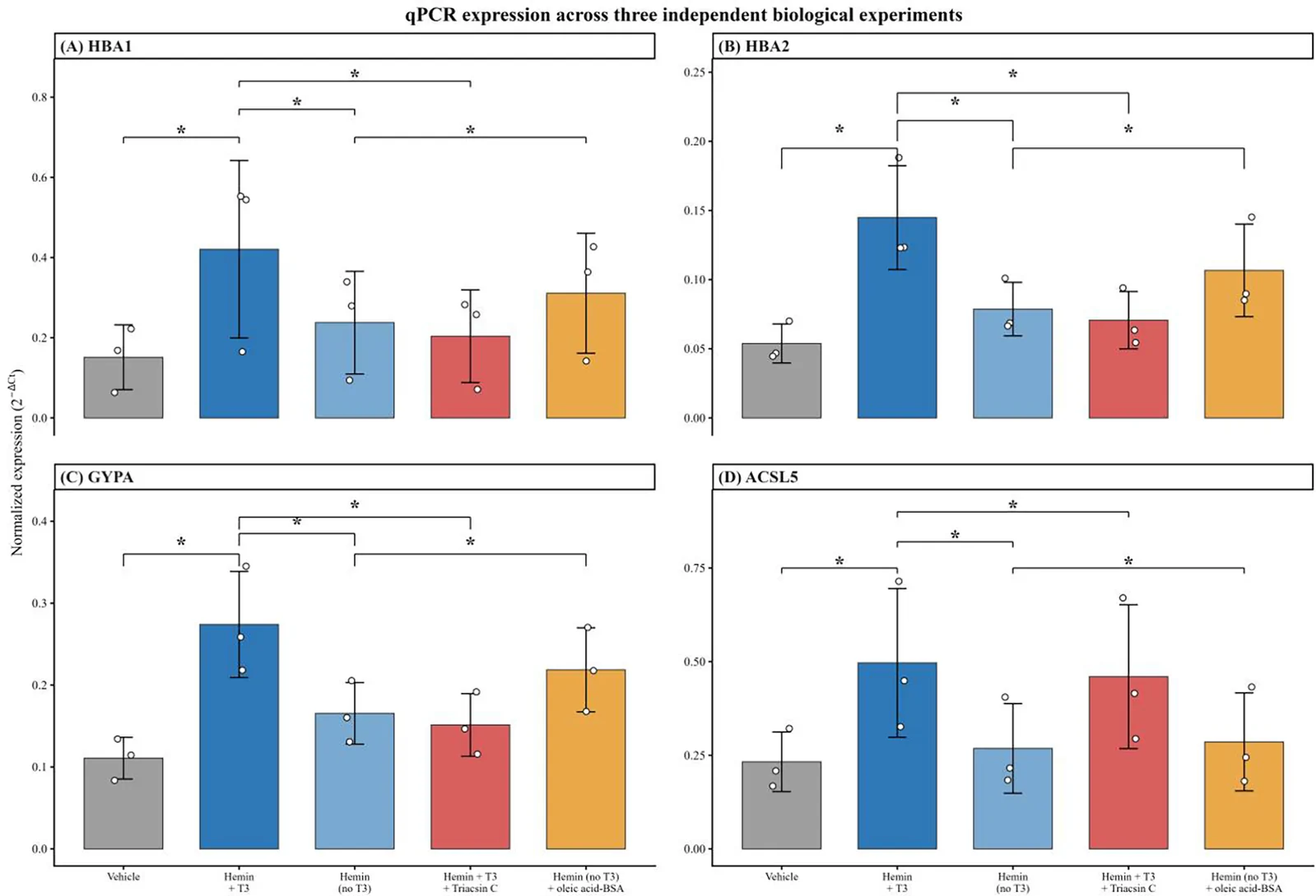
Bar-chart presentation with individual biological-replicate points of normalized qPCR expression across the five experimental conditions. **(A)** *HBA1*, **(B)** *HBA2*, **(C)** *GYPA*, and **(D)** *ACSL5*. Each bar represents the mean normalized expression from three independent biological experiments (*n* = 3), and error bars indicate the standard deviation. Within each biological experiment, three technical qPCR wells were averaged before calculation of the group mean. Expression was calculated as 2^−^ΔCt relative to *GAPDH*. Hemin plus T3 increased all four transcripts relative to vehicle, whereas omission of exogenous T3 reduced *HBA1*, *HBA2*, *GYPA*, and *ACSL5*. Triacsin C reduced erythroid-marker expression and produced a smaller reduction in *ACSL5* mRNA. Under the no-exogenous-T3 condition, oleic acid-BSA partially increased *HBA1*, *HBA2*, and *GYPA* while *ACSL5* mRNA remained stable.

Hemin plus T3 produced clear increases over vehicle: *HBA1* rose by 2.774 ± 0.399-fold (Holm-adjusted *p* = 0.0177), *HBA2* by 2.693 ± 0.062-fold (*p* = 0.00105), *GYPA* by 2.480 ± 0.192-fold (*p* = 0.00517), and *ACSL5* by 2.107 ± 0.145-fold (*p* = 0.0110). *ACSL5* rose in the same experiments and in the same direction as the three erythroid-marker transcripts.

The pattern reversed when exogenous T3 was omitted. Relative to hemin plus T3, *HBA1* fell to 0.566 ± 0.050-fold (Holm-adjusted *p* = 0.0177), *HBA2* to 0.544 ± 0.010-fold (*p* = 0.00105), *GYPA* to 0.605 ± 0.013-fold (*p* = 0.00240), and *ACSL5* to 0.537 ± 0.049-fold (*p* = 0.0222). This coordinated reduction was reproduced in each experiment and linked *ACSL5* expression to the T3-supported transcriptional state.

### Effects of pathway perturbations on erythroid-marker transcription

Triacsin C added to hemin plus T3 reduced *HBA1* to 0.472 ± 0.040-fold (Holm-adjusted, *p* = 0.0171), *HBA2* to 0.486 ± 0.038-fold (*p* = 0.00775), and *GYPA* to 0.551 ± 0.018-fold (*p* = 0.00449). *ACSL5* mRNA declined less, to 0.921 ± 0.018-fold (*p* = 0.0436). The larger changes in erythroid markers than in *ACSL5* transcript abundance are consistent with a pathway-level effect on long-chain acyl-CoA synthetase activity or downstream lipid handling.

Oleic acid-BSA was tested without exogenous T3. Relative to hemin plus vehicle, it increased *HBA1* by 1.357 ± 0.135-fold (Holm-adjusted *p* = 0.0356), *HBA2* by 1.342 ± 0.084-fold (*p* = 0.0136), and *GYPA* by 1.319 ± 0.037-fold (*p* = 0.00517). *ACSL5* expression changed little (1.061 ± 0.074-fold; *p* = 0.285). Added substrate shifted the erythroid markers partway toward the T3-supported pattern without a concomitant rise in *ACSL5* mRNA.

The whole-blood eQTL-SMR and K562 experiments addressed different biological questions. The former estimated a chronic genetically predicted expression association in a heterogeneous peripheral-blood mixture, whereas the latter measured an acute T3-responsive transcriptional state in a leukemia-derived erythroid model. Their opposing directions preclude a simple linear causal interpretation. The pathway perturbations instead identify long-chain fatty-acid activation and substrate availability as targets for direct mechanistic testing.

Values are mean ± standard deviation from three independent biological experiments and are expressed as 2^-^ΔCt normalized to *GAPDH*. Three technical wells were averaged within each experiment before group-level analysis; technical wells were not treated as independent observations. The means in this table form the bar heights and the standard deviations form the error bars in **Figure 4**.

## Discussion

The disease-level MR association was small and potentially affected by sample overlap, but the cross-biobank analyses reproduced the direction of the association. Methylation and expression SMR prioritized *ACSL5* and *ZDHHC6*, and the K562 experiments showed *ACSL5* responsiveness to T3 during hemin induction. The negative CpG-expression colocalization lookup and the absence of direct perturbation of *ACSL5* led us to disregard it as a candidate rather than an established mediator.

The provenance and scale of the primary MR result warrant attention (34). Both GWASs were UK Biobank-derived, and participant overlap may be substantial, but public summary-level metadata omit participant identifiers, detailed sampling frames, and the actual intersection. The extent of this overlap therefore cannot be quantified reliably. Sample overlap can bias two-sample MR toward a confounded observational association, particularly with weak instruments (35). The FinnGen broad hypothyroidism-to-UK Biobank anemia validation and the reciprocal UK Biobank hypothyroidism-to-FinnGen anemia analysis used different biobanks for exposure and outcome and reproduced the positive direction. This consistency makes sample overlap an unlikely sole explanation, although differences in phenotype definitions and association scales preclude direct coefficient comparison. Strict original-ID reverse MR was not estimable; the relaxed-threshold exploratory result was null and heterogeneous.

*ACSL5* was selected for follow-up because two of its CpGs passed Bonferroni correction with *p*_HEIDI_ > 0.01, its whole-blood expression provided an independent signal, its known function is relevant to lipid metabolism, and it was included in the available K562 experiments. *ZDHHC6* also received support at both molecular levels and remains a credible candidate, but no corresponding cellular experiment was available. These findings support prioritization rather than unique causality, and the absence of a CpG-expression colocalization pair limits locus-level mechanistic interpretation (29, 36).

K562 cells showed coordinated T3 responsiveness: hemin plus T3 increased *ACSL5* and the three erythroid-marker transcripts, whereas omission of exogenous T3 reduced all four. These transcriptional data establish neither a role for *ACSL5* in driving differentiation nor direct thyroid-hormone-receptor binding to an *ACSL5* regulatory element. Receptor ChIP, enhancer assays, receptor perturbation, and promoter analyses remain necessary to distinguish direct from indirect regulation. We therefore describe *ACSL5* as T3-responsive in this model.

The pharmacological contrast was informative at the pathway level. Triacsin C inhibits several long-chain acyl-CoA synthetase isoforms, whereas oleic acid-BSA broadly changes fatty-acid availability. The use of Triacsin C at 1 μmol/L was exploratory; dose–response, ACSL activity, and other target-engagement measurements were unavailable. The observed changes are consistent with altered long-chain fatty-acid activation or downstream lipid handling, but they cannot be assigned specifically to *ACSL5*. Oleic acid-BSA also shifted erythroid-marker expression without a corresponding change in *ACSL5* transcript abundance.

The downstream route remains unresolved. Long-chain acyl-CoA species may enter mitochondrial beta-oxidation, membrane phospholipid remodeling, triglyceride synthesis, or other pathways that influence redox balance, mitochondrial remodeling, substrate allocation, and heme/globin production (37–41). Distinguishing these possibilities will require measurements of lipid flux, acyl-CoA pools, mitochondrial respiration, membrane composition, and heme synthesis beyond the present qPCR data.

Standard clinical evaluation of anemia remains essential. The MR estimate supports attention to hemoglobin abnormalities in patients with hypothyroidism, while individual etiologies still require assessment for iron, vitamin B12, or folate deficiency, renal disease, inflammation, blood loss, and other causes (42, 43). Our results raise the possibility of an additional cell-intrinsic metabolic component for direct testing.

The primary MR included multiple estimators and standard sensitivity analyses, with source provenance and the unavailable participant intersection explicitly reported. Two forward validations used different biobanks for exposure and outcome. Reverse directionality was examined by swapping the original IDs at the prespecified genome-wide threshold and, after no instruments were available, in a labeled *post hoc* exploratory analysis. The molecular screen excluded the MHC and applied HEIDI, while the *post hoc* colocalization lookup tested the proposed CpG-expression connection. K562 findings were reproduced in three biological experiments, with technical wells averaged within each experiment.

The genetic and molecular analyses have several limitations. Both primary datasets were UK Biobank-derived, and public metadata do not disclose their exact participant overlap. Cross-biobank validation reduces concern about overlap as the sole source of the observed positive direction, although FinnGen and UK Biobank differ in phenotype definition and association scale. Strict original-ID reverse MR could not be estimated because the anemia exposure had no genome-wide-significant instruments. The *post hoc p* < 5 × 10^−6^ analysis used eight harmonized instruments, showed heterogeneity, and remains insufficient to exclude reverse causation. Most participants were of European ancestry; anemia definitions lacked etiologic subtypes; and the reconstructed *R*^2^ values were observed-scale estimates for a binary exposure. The two *ACSL5* CpGs and the *ACSL5* eQTL occupied distinct public colocalization groups, providing no positive evidence for a shared fine-mapped signal while leaving signal independence unresolved.

Experimental interpretation is also limited by the use of *GAPDH* as the sole reference gene. Although its Ct values were stable across the present conditions, future replication should use multiple reference genes whose stability has been validated in the erythroid experimental system.

The experimental evidence is also preliminary. It comes from three biological replicates in K562 cells and is restricted to mRNA readouts. This model captures an erythroid transcriptional component, not the systemic physiology of hypothyroidism-associated anemia. Future studies should include larger numbers of replicates, matched carrier and viability controls, multiple reference genes, amplification-efficiency data, and complete MIQE documentation (44). Protein abundance, enzyme activity, lipid metabolites, and direct differentiation phenotypes would be more informative than transcription alone.

An *ACSL5*-targeted siRNA knockdown experiment in the K562 erythroid model is being initiated, but no gene-specific experimental results are currently available. This experiment will test whether *ACSL5* is required for the T3-responsive erythroid transcriptional phenotype; rescue or controlled overexpression would still be needed to assess specificity and sufficiency. Thyroid-hormone-receptor ChIP, enhancer assays, or receptor perturbation would distinguish direct from indirect T3 regulation. Measurements of long-chain acyl-CoA pools, fatty-acid oxidation, phospholipid remodeling, heme synthesis, viability, and erythroid maturation would then identify the affected route. Replication in primary human CD34-positive erythroid cultures and functional follow-up of *ZDHHC6* should also be considered.

## Conclusion

*ACSL5* emerged as a T3-responsive candidate relevant to long-chain fatty-acid handling in an erythroid model. Cross-biobank analyses reproduced the disease-level direction, and the molecular-QTL results supported *ACSL5* follow-up. The CpG-expression colocalization result was negative, and the pharmacological interventions were not gene-specific. Direct *ACSL5* perturbation followed by validation in primary erythroid cells is the next step toward resolving the mechanism.

## Data Availability

The publicly available GWAS and molecular QTL datasets analyzed in this study are available from their original repositories. The primary GWAS datasets are available through OpenGWAS/MR-Base under the identifiers ebi-a-GCST90029022 and ukb-b-15790. Cross-biobank validation data were obtained from FinnGen, including finn-b-HYPOTHYROIDISM and FinnGen R12 D3_ANAEMIA. The molecular analyses used previously published blood mQTL resources, GTEx whole-blood eQTL data, and the publicly available Human Genotype-Phenotype Map (GPMap). The derived MR, SMR, and colocalization results, together with the raw qPCR Ct values, normalized expression data, and other data supporting the findings of this study, are provided within the article and its **Supplementary Material**. No additional participant-level data were generated in this study.
