# Supplementary material for: Cross-scale genetic, molecular, and cellular triangulation prioritizes ACSL5 as a T3-responsive candidate linking hypothyroidism liability to anemia
Source: Front Endocrinol (Lausanne). 2026 Oct 8;17:1943954. doi: 10.3389/fendo.2026.1943954 (PMC13686861; doi:10.3389/fendo.2026.1943954)
Supplement: Supplementary file 1 [file Supplementaryfile1.docx]

**Supplementary Material**

**Supplementary Figure S1.** SNP-specific and leave-one-out sensitivity analyses for the effect of genetically predicted hypothyroidism on anemia. (A) Forest plot showing the causal estimate and 95% confidence interval for each SNP, together with pooled estimates from the IVW and MR-Egger methods. (B) Leave-one-out analysis showing the IVW estimate after sequential exclusion of each SNP; the row labelled “All” represents the estimate obtained using the complete instrument set.

*This figure corresponds to the detailed SNP-level sensitivity plot moved from the main manuscript to the supplementary material to improve main-text readability.*
